# Early Pacemaker Implantation after Transcatheter Aortic Valve Replacement: Impact of PlasmaBlade™ for Prevention of Device-Associated Bleeding Complications

**DOI:** 10.3390/medicina57121331

**Published:** 2021-12-05

**Authors:** Alexander Lind, Majid Ahsan, Elif Kaya, Reza Wakili, Tienush Rassaf, Rolf Alexander Jánosi

**Affiliations:** Department of Cardiology and Vascular Medicine, West German Heart and Vascular Center Essen, University of Duisburg-Essen, 45147 Essen, Germany; Majid.AhsanEsfahani@uk-essen.de (M.A.); elif.kaya@uk-essen.de (E.K.); reza.wakili@uk-essen.de (R.W.); tienush.rassaf@uk-essen.de (T.R.); alexander.janosi@uk-essen.de (R.A.J.)

**Keywords:** bleeding, TAVR, anticoagulation, pacemaker, clopidogrel, antiplatelet therapy, DAPT, PlasmaBlade

## Abstract

*Background and Objectives*: Permanent pacemaker implantation (PPI) is frequently required following transcatheter aortic valve replacement (TAVR). Dual antiplatelet therapy (DAPT) or oral anticoagulation therapy (OAK) is often necessary in these patients since they are at higher risk of thromboembolic events due to TAVR implantation, high incidence of coronary artery diseases (CAD) with the necessity of coronary intervention, and high rate of atrial fibrillation with the need of stroke prevention. We sought to evaluate the safety, efficiency, and clinical outcomes of early PPI following TAVR using the PlasmaBlade™ (Medtronic Inc., Minneapolis, MN, USA) pulsed electron avalanche knife (PEAK) for bleeding control in patients under DAPT or OAK. *Materials and Methods*: This retrospective single-center study included patients who underwent PPI after transfemoral TAVR (TF) at our center between December 2015 and May 2020. All PPI were performed using the PlasmaBlade™ Device. *Results*: The overall PPI rate was 14.1% (83 of 587 patients; 82.5 ± 4.6 years; 45.8% male). The PPI procedures were used to treat high-grade atrioventricular block (81.9%), severe sinus node dysfunction (13.3%), and alternating bundle branch block (4.8%). At the time of the procedure, 35 (42.2%) patients received DAPT, and 48 (57.8%) patients received OAK (50% with vitamin K antagonist (VKA) and 50% with novel oral anticoagulants (NOAK)). One device-pocket hematoma treated conservatively occurred in a patient (1.2%) receiving NOAK. Two re-operations were necessary in patients due to immediate lead dislocation (2.4%). *Conclusions*: The results of this study illustrate that the use of PlasmaBlade™ for PPI in patients after a TAVR who require antithrombotic treatment is feasible and might result into lower rates of severe bleeding complications compared to rates reported in the literature. Use of the PlasmaBlade device may be considered in this specific group of patients because of their high risk of bleeding.

## 1. Introduction

Aortic valve stenosis (AS) is the most frequent valvular disease in the elderly population. Transcatheter aortic valve replacement (TAVR) is an effective treatment for severe symptomatic AS in high-risk patients [1], and its use has rapidly increased worldwide in recent years. Excellent results from clinical trials have initiated the reassessment of the recommended aortic stenosis treatments and may prompt a wider use of TAVR. Conduction disturbances (CDs), such as high-degree atrioventricular block (AVB) due to balloon valvuloplasty or self-expanding forces of the valve requiring permanent pacemaker implantation (PPI), are the most common complications after TAVR. PPI incidence is reportedly 2–51% [2,3,4] with variations across studies and valve types with important clinical implications. PPI after TAVR is often more challenging due to patients’ characteristics, such as frailty, age >75 years, high burden of comorbidities as coronary artery disease (CAD), the administration of antithrombotic agents periprocedural, increased bleeding risk due to gastrointestinal angiodysplasia, and change in factor VIII coagulant activity (Heyde’s syndrome) [5,6].

Single antiplatelet was recently recommended as a standard treatment option after TAVR in patients without the need for oral anticoagulation [7]. However, CAD is present in up to 50% in the elderly TAVR population with a need for percutaneous coronary intervention (PCI) of coronary artery stenosis >70% in proximal segments during the pre-TAVR work-up [8]. This leads to the need for DAPT with increased risk for the development of a device-pocket hematoma which is associated with an increased risk of infection, delayed mobilization and prolonged hospitalization in the high-risk elderly population [6].

Device-pocket hematoma is a common complication after PPI, especially in patients receiving anticoagulation therapy (OAK) and/or DAPT (reported rates of 2–5%) [4,9,10]. DAPT increases the risk of a bleeding complication five-fold regardless of 30% non-responder rate in patients under clopidogrel and aspirin. Under so-called triple therapy, the risk can be as high as 40% [11]. The risk of device-pocket hematoma with heparin bridging is reported with 17–31% [11,12,13]. Continuation of OAK during PPI is associated with an incidence of pocket bleeding of 2–7% [14].

The timing of PPI in the context of anticoagulation is controversial. CDs may be transient or have delayed onset [10,11,12,13]. Many centers started performing PPI soon after TAVR at the increased risk of bleeding complications related to dual antiplatelet therapy (DAPT) or the combination of DAPT and heparin bridging therapy [6].

To prevent bleeding complications, different strategies are being pursued including specific antithrombotic regimens and surgical considerations. In this context, the PlasmaBlade™ pulsed electron avalanche knife (PEAK) is a low-thermal-injury surgical instrument for soft-tissue cutting that uses brief precise pulses of radiofrequency energy. The PlasmaBlade™ controls bleeding while inflicting less tissue injury and causing minimal scar formation [15,16]. The PEAK Surgery System has a wide range of capabilities, and its hemostatic capability can be increased to a level equivalent to that of conventional electrosurgical technology with less thermal injury.

The present study aimed to evaluate the safety and efficiency using the PlasmaBlade™ for prevention of bleeding complications in TAVR patients under DAPT or OAK-Therapy requiring early or delayed PPI (<48 h or >48 h after TAVR) to identify parameters leading to increased morbidity and prolonged hospitalization.

## 2. Materials and Methods

Patients and data collection: Between December 2015 and May 2020, 587 patients underwent TF-TAVR at our center. This retrospective single-center observational study enrolled 83 (14.1%) consecutive patients who underwent PPI after TAVR. Routinely collected data were recorded to evaluate the management safety and efficiency in all patients. TAVR-related PPI was defined as PPI at ≤30 days after the procedure.

The study was performed in accordance with the Declaration of Helsinki, and the study protocol was approved by the ethics committee of the Faculty of Medicine of the University of Duisburg-Essen (No. 16–6894-BO). Written informed consent was obtained from each patient. All parameters were analyzed anonymously. All patients were diagnosed with severe symptomatic AS. Patients who underwent previous atriovenous surgery (replacement/repair) or TAVR (valve-in-valve procedure) and had a previously implanted pacemaker, implantable cardiac defibrillator, or cardiac resynchronization therapy were excluded from the study.

Pacemaker implantation procedures: PPI was performed in accordance with the European Society of Cardiology guidelines [17] for cardiac pacing and indicated either for third-degree or advanced second-degree AVB at any anatomical level that was not expected to resolve or for sinus node dysfunction and documented symptomatic bradycardia. Implantation timing was determined individually for each patient. The selection of a single- or dual-chamber device was decided at the discretion of the implanter in accordance with the European Society of Cardiology guidelines. All patients received intravenous antibiotic prophylaxis before the procedure according to the European Society of Cardiology guidelines [18]. PPI was performed by expert cardiac electrophysiologist under local anesthesia essentially as described previously [16,19]. All procedures were performed using the PlasmaBlade™ (Medtronic Inc., Minneapolis, MN, USA) in cutting mode 6 and coagulation mode 8. Conventional electrocautery was not used. Antiplatelet therapy was continued. The standard procedure involved access to the cephalic vein. Leads were placed under fluoroscopic guidance. Tight banding was performed in all patients for 24 h after the procedure to reduce the rate of bleeding and lead detachment.

TAVR implantation procedures: TAVR patients were selected by our local heart team, which comprised interventional cardiologists, cardiac surgeons, and cardiovascular anesthesiologists. TAVR was performed by a multidisciplinary heart team in a hybrid operating room using the standard technique [20,21] with patients under conscious sedation [22,23,24,25] and percutaneous femoral artery access and closure. One of the two bioprostheses with a current Conformité Européenne mark approval (SAPIEN S3 (Edwards Lifesciences, Irvine, CA, USA) and CoreValve Evolut R (Medtronic Inc., Minneapolis, MN, USA)) were implanted. All patients were periprocedural, were monitored using six-electrode virtual 12-lead electrocardiography and pulse oximetry, and were routinely transferred to the intensive care unit after the procedure for post-interventional monitoring for a minimum of 24 h. Vital signs were continuously monitored, with special attention paid to identifying cardiac rhythm disturbances, neurological disorders, and access-site complications and to assessing systemic blood pressure and fluid balance. 

Unfractionated heparin was administered during the procedure. The initial heparin dose was 70 U/kg, and the activated clotting time (ACT) was measured last before valvuloplasty or the insertion of the valve. If it was >250 s, an additional heparin bolus was administered.

Study definitions: The study population was divided into two groups: patients on DAPT containing aspirin and clopidogrel due to PCI before TAVR (DAPT-group) and patients with the need for OAK and single clopidogrel therapy after TAVR (OAK-group). Four patients on triple therapy needing DAPT due to previous PCI and OAK were included in the OAK group.

Median duration from TAVR to PPI was 2 days. Therefore, we defined two similar sized groups of patients with conduction disturbances (CDs) requiring “early PPI” (within 48 h) vs. “late PPI” (after 48 h).

Anticoagulation before TAVR: In patients that were on Vitamin-K-Antagonist (VKA) before, TAVR anticoagulation was paused until the International Normalized Ratio (INR) of 2.0 was reached. If necessary, bridging with intravenous (i.v.) full-dose unfractionated heparin (FDUH) was started before TAVR when INR was below 2.0. Heparin was paused 6 h before TAVR. Novel Oral Anticoagulants (NOACs) were stopped at least 48 h before the TAVR and resumed on the day after the procedure. 

Anticoagulation after TAVR: If PCI was performed before TAVR, DAPT was continued for up to 6 months post PCI and thereafter reduced to single antiplatelet therapy (SAPT) containing aspirin only lifelong. In patients without previous PCI, a loading dose of clopidogrel (600 mg per os) was administered after completion of the TAVR procedure and continued for 6 months according to the 2017 guideline recommendations. Patients with the need for OAK and new evidence of CAD and PCI before TAVR continued on OAK and DAPT for 4 weeks. Bridging with FDUH was resumed on the first day after TAVR. VKA was simultaneously started. NOAC was re-initiated on the first postoperative day. Thereafter the anticoagulation regime was reduced to lifelong OAK and single platelet inhibition for 5 more months.

Anticoagulation during and after PPI: If treatment with Vitamin K antagonist (VKA) after TAVR was still interrupted and early PPI (<48 h) was necessary, patients received FDUH bridging therapy on the first day after TAVR up to 6 h before PPI. If VKA was already resumed in patients with late CDs and necessity of PPI (>48 h), VKA was continued with target INR between 2.0 and 3.0 on the day of PPI.

In patients with early PPI, FDUH was reinitiated 24 h after PPI and continued until a therapeutic INR was achieved. Novel oral NOACs were stopped at least 48 h before the PPI procedure and were restarted 48 h after the procedure. 

Endpoint definition: Peri- and postprocedural complications were evaluated according to the Valve Academic Research Consortium 2 (VARC-2) [26] and Bleeding Academic Research Consortium (BARC) definitions [27]. (Supplementary Table S1)

Statistical analysis: Procedural data, including demographic and outcome data, were entered into a database. Statistical analyses were performed using SPSS version 24.0 (IBM Corp., Armonk, NY, USA). Continuous variables are expressed as mean and standard deviation, whereas categorical variables are presented as number and percentage. For normally distributed variables, intergroup comparisons were performed using Student’s *t*-test for continuous variables and the χ2 test for categorical variables. With regard to the non-normally distributed continuous variables, the groups were compared using the Mann–Whitney U-test. For all analyses, *p* values < 0.05 were considered statistically significant.

## 3. Results

Baseline demographics of the study population: The patients’ baseline characteristics are listed in Table 1. Our study cohort represents a typical transfemoral TAVR population with severe symptomatic AS (mean aortic pressure gradient 42.7 ± 20.1 mmHg) and high operative risk due to age and comorbidities (EuroScore 17.6 ± 11.7%, STS-Score 4.3 ± 2.4%). A total of 83 TAVR patients (mean age, 82.5 ± 4.6 years; 45.8% male) were included. Most of the patients were in the New York Heart Association (NYHA) classification III/IV (*n* = 74, 89.2%). Coronary artery disease was documented in 56 patients (67.5%). Seven patients (8.4%) had previous coronary artery bypass grafting. PCI within 6 months before TAVR was performed in 23 (27.7%) patients.

Mean ejection fraction was 52.4 ± 9.8%. A total of 44 patients (53.0%) had a history of atrial fibrillation, whereas previous cerebrovascular events were present in 4 patients (4.8%). Peripheral arterial disease (PAD) was present in 22 patients (26.5%), and cerebral vascular disease was present in 31 patients (37.3%). A history of diabetes was present in 26 patients (31.3%). Impaired renal function defined as GFR < 60 mL/min/1.73 m^2^ was diagnosed in 15 patients (29.4%), and mean GFR was 54.5 mL/min/1.73 m^2^ ± 21.2 mL/min/1.73 m^2^ [28].

These parameters did not differ significantly between the DAPT-group containing patients on aspirin and clopidogrel and the NOAK-group containing patients on OAK and clopidogrel. Only atrial fibrillation was significantly more present in the OAK-group than in the DAPT-group (Table 1).

Indication for Pacemaker: The leading indication for PPI was new complete atrioventricular block (AVB) (79.55%). Sick sinus syndrome (SSS) with prolonged pauses and slow atrial fibrillation was present in 8 patients (9.6%) and 4 patients (4.8%), respectively. One patient (1.2%) had 1st degree AVB + left bundle brunch block. Two patients developed 2nd degree AVB and trifascicular block (2.4%). Single- and dual-chamber devices were implanted in 27 (31.3%) and 55 (66.3%) patients, respectively. Two patients (2.4%) received three-chamber-ICD devices due to severe heart failure with reduced ejection fraction and severe coronary artery disease (Table 2).

**Figure 1:**
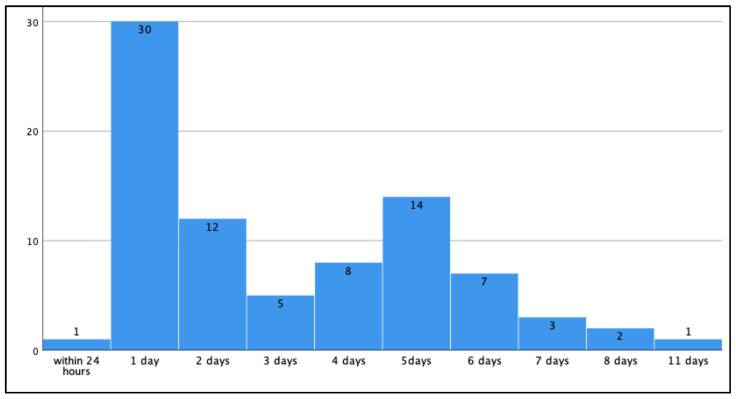
Timing of permanent pacemaker implantation (PPI) after transcatheter aortic valve replacement (TAVR).

Comparison of clinical parameters in patients with respect to PPI timing (48 h vs. later): 43 of 83 patients (51.8%) underwent early PPI (within 48 h). Within this group, the implantation of a permanent pacemaker was performed on the day of TAVR implantation in one patient and on the first or second postinterventional day in 30 vs. 12 patients, respectively. Late PPI took place in 40 patients up to 11 days post-intervention (early PPI 1.3 ± 0.45 days vs. late PPI 5.2 ± 1.6 days, *p* = 0.0). (Figure 1, Table 3).

Multiple clinical parameters were analyzed with respect to differences between early (within 48 h) and late PPI (>48 h) after TAVR (Table 3). Length of postoperative hospital stay did not differ in patients with early compared to late PPI (7.2 ± 3.5 vs. 9.88 ± 9.1, *p* = 0.086). A significant difference was only seen in patient’s total hospital stay. This was significantly longer in patients with late PPI (21.0 ± 9.5 vs. 14.3 ± 5.3; *p* ≤ 0.001). Other parameters like total procedure time, GFR, renal insufficiency, age, logistic EuroScore and STS-Score did not differ. Only one clinically relevant device-pocket hematoma was seen in a patient with early PPI with interrupted VKA who received bridging therapy with intravenous heparin. No further perioperative complications were detected in patients with early PPI under dual-antiplatelet therapy and anticoagulation.

Procedural characteristics of the study population comparing DAPT-group to OAK-group: Table 4 lists the procedural characteristics of the study population and both groups. The total procedure time (time from the first skin incision until the end of surgery) was similar in both groups (37.9 ± 14.1 vs. 39.2 ± 17.3, *p* = 0.713). However, the postoperative and total hospital stay was longer in the OAK-group compared to the DAPT group (6.3 ± 2.9 vs.10.0 ± 8.4, *p* = 0.006 and 14.8 ± 6.1 vs. 19.5 ± 9.2, *p* = 0.011, respectively). 

Anticoagulation regimes in the DAPT-group compared to the OAK-group: Nearly all patients (*n* = 82, 98.8%) underwent PPI with clopidogrel due to post-TAVR loading. Of these patients (*n* = 35), 42.2% were on DAPT consisting of aspirin and clopidogrel during PPI due to previous PCI (DAPT-group). More than half of the patients (*n* = 48; 57.8%) had an additional indication for oral anticoagulation in combination with single antiplatelet therapy consisting of clopidogrel post TAVR (OAK-group). Only four patients within the OAK-group (4.8%) received triple anticoagulation due to previous implantation of coronary stents. Exactly half of the patients were treated with VKA (*n* = 24; 50.0%), and the other half received a NOAC. Within the NOAC group, apixaban (*n* = 14; 58.3%) was most frequently used, whereas rivaroxaban (*n* = 6; 25%), edoxaban (*n* = 3; 12.5%), and dabigatran (*n* = 1; 4.2%) were used less often (Table 4). 

Complications and coagulation status within the DAPT-group compared to the OAK-group: Table 5 lists the complications in both study groups. One patient (1.20%) in the OAK-group developed a device-pocket hematoma which could be treated conservatively. The hematoma developed 72 h after PPI during bridging therapy with intravenous heparin VKA therapy being interrupted. This did not differ from the DAPT-group although the HAS-BLED Score was higher in the OAK-group compared to the DAPT-group (4.0 ± 0.9 vs. 3.5 ± 0.6, *p* = 0.002). The loss of Hb > 2 mg/dL did not differ in the DAPT compared to the OAK-group (5.7% vs. 6.3%, *p* = 0.919), and consequently, BARC bleeding events did not differ in both groups. Two re-operations were necessary in patients due to immediate lead dislocation. (2.41%). There were no cases of hemothorax. No patient died during the first 30 days.

## 4. Discussion

Early complications of PPI, even when performed by an experienced team of cardiologists or surgeons, are common. Elderly and extremely fragile patients may be at increased risk of implant complications.

Concerning our cohort, it is important to emphasize that DAPT therapy is not recommended anymore on a regular base in current guidelines after TAVR [29,30]. However, CAD is present in up to 50% of the TAVR population [31] leading to the need of PCI in 16% to 34% of TAVR patients before TAVR implantation [32]. This is in line with our study showing a PCI rate of 27.7% before TAVR. The reported risk of device pocket hematoma after PPI with DAPT therapy ranges from 0.7% to 24% [9,33,34]. Contrary to these results we saw no severe bleeding complication in the DAPT group.

Additionally, TAVR patients have multiple comorbidities, and atrial fibrillation is very common in the elder patients with a prevalence of up to 38% [35]. The NOACs dabigatran, rivaroxaban, apixaban and edoxaban are increasingly prescribed in atrial fibrillation patients, although dosage in elderly patients, food- and drug-interactions, laboratory tests for monitoring, and antidote are not clarified [36]. In our cohort NOAC was present in 50% of patients in the OAK group. In combination with chronic kidney disease, being present in 50.6% of our elder TAVR cohort, NOACS are well known having increased active substance levels negatively impacting the survival of older adults treated with PPI [37]. 

To reduce bleeding complications under NOAC therapy especially in patients with impaired renal function interruption of NOAC for 24 h–48 h before PPI is recommended [38]. In our study, we interrupted NOACs for 48 h but still recognized one severe bleeding complication in a patient with an impaired renal function under NOAC therapy. Therefore, timing of PPI is crucial to control for bleeding complications in patients with impaired renal function and NOAC.

Patients receiving VKA therapy comprise another high-risk cohort. As PPI with continuation of warfarin therapy in TAVR patients is not possible, heparin-bridging therapy is necessary. However, it is associated with an increased risk of bleeding with a prevalence of up to 20% for device-pocket hematoma versus 2–4% in patients in whom warfarin was persistently used [17,19,20,25,39]. In some studies, heparin-bridging therapy was reported to pose an even higher risk than DAPT [40]; therefore, the continuation of warfarin therapy during PPI has been favored [39], but this is not possible during TAVR. Continuous multiple anticoagulation therapies are possible, but patients with high HAS-BLED scores or valvular heart disease require careful attention during PPI [41]. HAS-BLED score in our study was 3.8 ± 0.8 underlining the need for sufficient anticoagulation before and after TAVR and therefore increasing the risk for PPI-related complications like device pocket hematoma. 

Whether a device-pocket hematoma is a risk factor for PPI-related infections remains controversial [9,10]. However, device-pocket hematoma undoubtedly is an inconvenient complication associated with pain, especially in elderly patients at high risk owing to a prolonged recovery, which leads to pocket infections and wound dehiscence. Furthermore, intraoperative bleeding is related to prolonged procedure time, the increased risk of infection, and prolongation of hospitalization and treatment costs [40].

In our study, the overall perioperative complication rate and overall incidence of a clinically significant device-pocket hematoma were both 1.2%, whereas we considered only hematomas with clinical impact, namely, those determining prolonged hospitalization or requiring surgical reintervention. Even in the group comprising patients who underwent early PPI within 48 h after TAVR, no cases of bleeding complications were observed.

The low complication rate may be due to all surgeries being performed by an experienced operator using PlasmaBlade™. PlasmaBlade™ is a novel surgical tool that uses brief (40-ms range) radiofrequency pulses to induce electrical plasma along the edge of a 12.5 mm thin insulated electrode, allowing it to operate at low temperatures in the range of 40–170 °C. Standard cautery instruments are widely available and operate at high temperatures in the range of 200–350 °C. This creates an effective cutting edge while the blade stays near body temperature resulting in an effective bleeding control with less thermal tissue injury and damage. Furthermore, it provides atraumatic, scalpel- like cutting precision and electrosurgical-like hemostasis, while acute thermal injury depth is reduced by 74% [16,42,43,44]. PlasmaBlade™ incisions demonstrated reduced inflammatory response and scar width in healing skin compared with conventional electrocautery or scissors and reducing bleeding complications significantly (59%) [15,44]. Therefore, data support the use of the PlasmaBlade™ in patients undergoing PPI [16,45].

Despite of the above-mentioned advantages of the PlasmaBlade™, the acquisition costs of the PlasmaBlade™ are much higher than those of a conventional electrocautery unit. Further data demonstrating a reduction in the overall complication rate, procedure time, and length of hospital stay in TAVR patients which might translate into cost savings are required to establish PlasmaBlade™ as an alternative to conventional electrocautery unit. Until now, there are only scarce data demonstrating significantly reduced procedure time, length of hospital stay and cost effectiveness using PlasmaBlade^TM^ in patients undergoing pacemaker device replacement [46,47].

Therefore, our study is one of the first addressing this issue and highlighting the potential benefit of such a novel approach in TAVR patients requiring blood-thinning therapy.

## 5. Conclusions

Our findings suggest that PPI after TAVR using PEAK PlasmaBlade™ is per se safe and is not associated with an increase of peri-procedural bleeding events in TAVR-Patients on DAPT or OAK therapy. Further studies comparing PlasmaBlade™ and conventional electrocautery are warranted to evaluate whether PlasmaBlade™ is superior to conventional electrocautery for post-TAVR-PPI.

## 6. Limitations

The present study is a single-center retrospective observational report with potential methodology-inherent bias that is common to this study type. Considering the lack of a control group under traditional electrosurgery, it is not possible to conclude that this approach is safe per se, despite the low perioperative complication rate. Due to the retrospective design of the study, we are not able to define the loss of documentation of minor complications. Since our study included a special patient cohort, the number of included patients was small (13.4% of the overall cohort), which leads to a hypothesis-generating conclusion. Nevertheless, we could demonstrate the feasibility and high safety in the studied high-bleeding-risk cohort of patients undergoing PPI after TAVR using of the PlasmaBlade™ device.

## Figures and Tables

**Table 1:** Baseline demographic and clinical characteristics of the study population (DAPT vs. OAK).

Variables	Overall (*n* = 83)	DAPT-Group (*n* = 35)	OAK-Group (*n* = 48)	*p*-Value
Age (years)	82.5 ± 4.6	82.3 ± 4.9	82.7 ± 4.4	0.791
Male patients, n (%)	38 (45.8)	14 (40.0)	24 (50.0)	0.367
Body mass index (kg/m^2^), ±SD	27.2 ± 5.0	27.1 ± 5.4	27.3 ± 4.8	0.442
NYHA III/IV, *n* (%)	74 (89.2)	30 (85.7)	44 (91.7)	0.389
Coronary artery disease, n (%)	56 (67.5)	25 (71.4)	31 (64.6)	0.511
PCI within 6 months before TAVR, *n* (%)	23 (27.7)	14 (40.0)	9 (18.8)	0.059
Previous coronary artery bypass graft, *n* (%)	7 (8.4)	4 (11.4)	3 (6.3)	0.401
Left ventricular ejection fraction (%), ±SD	52.4 ± 9.8	52.9 ± 9.4	52.0 ± 10.2	0.719
History of atrial fibrillation, *n* (%)	44 (53)	2 (5.7)	42 (87.5)	<0.001
Previous cerebrovascular event, *n* (%)	4 (4.8)	0	4 (8.3)	0.134
Peripheral vascular disease, *n* (%)	22 (26.5)	11 (31.4)	11 (26.5)	0.538
Cerebral vascular disease, *n* (%)	31 (37.3)	14 (40.0)	17 (35.4)	0.844
Diabetes, n (%)	26 (31.3)	7 (20.0)	19 (39.6)	0.097
Renal insufficiency (GFR < 60 mL/min/m^2^), n (%)	42 (50.6)	16 (45.7)	26 (52.2)	0.590
GFR (ml/min/m^2^), ±SD	54.4 ± 21.2	56.7 ± 17.9	52.7 ± 23.3	0.381
Logistic EuroScore (%), ±SD	17.6 ± 11.7	15.7 ± 11.2	19.0 ± 11.97	0.210
Society of Thoracic Surgeons score (%), ±SD	4.3 ± 2.4	3.9 ± 2.2	4.6 ± 2.5	0.282
Aortic Valve Area (cm^2^), ±SD	0.7 ± 0.2	0.7 ± 0.1	0.7 ± 0.2	0.374
Mean Aortic Pressure Gradient (mmHg), ±SD	42.7 ±20.1	41.9 ± 11.9	43.3 ± 24.5	0.764

Data are presented as mean ± standard deviation (SD) or number (%). PCI = percutaneous coronary intervention, NYHA = New York Heart Association, GFR = Glomerular filtration rate.

**Table 2:** Indication for pacemaker implantation and device type.

Indication for Pacemaker	
Complete AVB	66 (79.5)
Slow AF	4 (4.8)
SSS/tachy-brady syndrome/prolonged pauses	8 (9.6)
1st degree AVB + LBBB	1 (1.2)
2nd degree AVB	2 (2.4)
Trifascicular block	2 (2.4)
Device Type	
Single-chamber device	26 (31.3%)
Dual-chamber device	55 (66.3%)
Three-chamber device	2 (2.4%)

Data presented as number (%). AVB = atrioventricular block, AF = atrial fibrillation, SSS = sick sinus syndrome, LBBB = left bundle-branch block. Data presented as number (%).

**Table 3:** Comparison of clinical parameters in patients with respect to PPI timing (48 h vs. later).

Variables	Overall (*n* = 83)	Early PPI (*n* = 43)	Late PPI (*n* = 40)	*p*-Value
Age (years)	82.5 ± 4.6	82.58 ± 4.5	82.5 ± 4.8	0.956
Male patients	38 (45.8)	19 (61.3)	19 (36.5)	0.943
Time to PPI after TAVR (days)	3.2 ± 2.3	1.3 ± 0.45	5.2 ± 1.6	<0.001
Total procedure time (min)	38.7 ± 15.96	41.0 ± 16.6	36.0 ± 14.8	0.153
Length of postoperative hospital stay (days)	8.5 ± 6.9	7.19 ± 3.5	9.9 ± 9.1	0.086
Total hospital stay (days)	17.5 ± 8.3	14.26 ± 5.3	21.0 ± 9.5	<0.001
Renal insufficiency (GFR < 60 mL/min/m^2^)	42 (50.6)	20 (64.5)	22 (42.3)	0.580
GFR (ml/min/m^2^)	54.4 ± 21.2	56.95 ± 22.6	51.7 ± 19.4	0.261
Logistic EuroScore (%)	17.6 ± 11.7	16.98 ± 8.6	18.3 ± 14.1	0.618
Society of Thoracic Surgeons score (%)	4.3 ± 2.4	4.9 ± 2.95	3.8 ± 1.6	0.109

Data are presented as mean ± standard deviation or number (%); GFR = Glomerular filtration rate.

**Table 4:** Procedural characteristics and coagulation regimes of the study population (DAPT therapy vs. OAK therapy).

Patients	*n* = 83	DAPT-Group *n* = 35 (42.2%)	OAK-Group *n* = 48 (57.8%)	*p*-Value
Procedure characteristics				
Total procedure time (min)	38.7 ± 16.0	37.9 ± 14.1	39.2 ± 17.2	0.734
Length of postoperative hospital stay (days)	8.5 ± 6.9	6.3 ± 2.9	10.0 ± 8.4	0.006
Hospital length of stay (Days)	17.5 ± 8.3	14.8 ± 6.1	19.5 ± 9.2	0.011
CRP before implant (mg/dL)	4.3 ± 3.5	3.5 ± 2.9	5.1 ± 4.0	0.059
Anticoagulation				
Clopidogrel	82 (98.8)	35	47	0.391
Dual-antiplatelet therapy	35 (42.2)	35		
Triple Therapy	4 (4.8%)		4	
Oral Anticoagulation	48 (57.8)	0	48	
VKA	24 (50)	0	24	
NOAC	24 (50)	0	24	
Rivaroxaban 20 mg q.d.			6 (7.2)	
Edoxaban 60 mg q.d.			3 (3.6)	
Apixaban 5 mg b.i.d.			14 (16.9)	
Dabigatran 150 mg b.i.d.			1 (1.2)	

Data are presented as mean ± standard deviation (SD) or number (%). VKA = Vitamin K antagonist, NOAC = Novel oral anticoagulant, q.d. = once a day, b.i.d. = twice a day.

**Table 5:** Complications and coagulation status in the DAPT-group compared to the OAK-group.

Patients	*n* = 83	DAPT-Group *n* = 35 (42.2%)	OAK-Group *n* = 48 (57.8%)	*p*-Value
Coagulation Status				
INR at implant	1.2 ± 0.3	1.1 ± 0.7	1.2 ± 0.3	0.003
HAS-BLED score	3.8 ± 0.8	3.5 ± 0.6	4.0 ± 0.92	0.002
Complications				
Device Pocket Hematoma	1 (1.2)	0	1 (2.1)	
Loss of >2 mg/dL Hb before and after PPI	5 (6.0)	2 (5.7)	3 (6.3)	1.0
Difference of Hb before and after PPI (mg/dL)	0.3 ± 1.1	0.4 ± 0.9	0.2 ± 1.3	0.492
BARC Type 0	78 (94.0)	33 (39.8)	45 (54.2)	0.661
BARC Type 2	4 (4.8)	2 (5.7)	2 (4.2)	
BARC Type 3	1 (1.2)	0	1 (2.1)	
Re-Operation due to lead dislocation	2 (2.4)	1 (2.9)	1 (2.1)	0.823

Data are presented as mean ± standard deviation (SD) or number (%). NOAC = Novel oral anticoagulant, INR = International normalized ratio, Hb = Hemoglobin, BARC = Bleeding Academic Research Consortium.

## Data Availability

The data presented in this study are available on request from the corresponding author.

## Supplementary Materials

The following are available online at https://www.mdpi.com/article/10.3390/medicina57121331/s1.
