# Supplementary material for: Early Pacemaker Implantation after Transcatheter Aortic Valve Replacement: Impact of PlasmaBlade™ for Prevention of Device-Associated Bleeding Complications
Source: Medicina (Kaunas). 2021 Dec 5;57(12):1331. doi: 10.3390/medicina57121331 (PMC8707306; doi:10.3390/medicina57121331)
Supplement: Supplementary file 1 [file medicina-57-01331-s001.zip › medicina-1423848-supplementary.pdf]

Supplemental Table S1. VARC-2 Criteria.

TIA and Stroke	
Stroke	duration of a focal or global neurological deficit ≥24 h; OR <24 h if available neuroimaging documents a new haemorrhage or infarct; OR the neurological deficit results in death
TIA	duration of a focal or global neurological deficit <24 h, any variable neuroimaging does not demonstrate a new haemorrhage or infarct
Disabling stroke:	an mRS score of 2 or more at 90 days and an increase in at least one mRS category from an individual's pre-stroke baseline
Non-disabling stroke	an mRS score of <2 at 90 days or one that does not result in an increase in at least one mRS category from an individual's pre-stroke baseline
Bleeding	
Life-threatening or disabling bleeding	Fatal bleeding (BARC type 5) OR Bleeding in a critical organ, such as intracranial, intraspinal, intraocular, or pericardial necessitating pericardiocentesis, or intramuscular with compartment syndrome (BARC type 3b and 3c) OR Bleeding causing hypovolaemic shock or severe hypotension requiring vasopressors or surgery (BARC type 3b) OR Overt source of bleeding with drop in haemoglobin ≥5 g/dl or whole blood or packed red blood cells (RBCs) transfusion ≥4 units (BARC type 3b)
Major bleeding (BARC type 3a)	Overt bleeding either associated with a drop in the haemoglobin level of at least 3.0 g/dl or requiring transfusion of two or three units of whole blood/RBC, or causing hospitalization or permanent injury, or requiring surgery AND Does not meet criteria of life-threatening or disabling bleeding
Minor bleeding (BARC type 2 or 3a, depending on the severity)	Any bleeding worthy of clinical mention (e.g. access site haematoma) that does not qualify as life-threatening, disabling, or major
Acute kidney injury (AKNI classification)	
Stage 1	Increase in serum creatinine to 150–199% (1.5–1.99 × increase compared with baseline) OR increase of ≥0.3 mg/dl (≥26.4 mmol/l) OR Urine output <0.5 ml/kg/h for >6 but <12 h
Stage 2	Increase in serum creatinine to 200–299% (2.0–2.99 × increase compared with baseline) OR Urine output <0.5 ml/kg/h for >12 but <24 h
Stage 3	Increase in serum creatinine to ≥300% (>3 × increase compared with baseline) OR serum creatinine of ≥4.0 mg/dl (≥354 mmol/l) with an acute increase of at least 0.5 mg/dl (44 mmol/l) OR Urine output <0.3 ml/kg/h for ≥24 h OR Anuria for ≥12 h
Vascular access site and access-related complications	
Major vascular complications	Any aortic dissection, aortic rupture, annulus rupture, left ventricle perforation, or new apical aneurysm/pseudo-aneurysm OR Access site or access-related vascular injury (dissection, stenosis, perforation, rupture, arterio-venous fistula, pseudoaneurysm, haematoma, irreversible nerve injury, compartment syndrome, percutaneous closure device failure) leading to death,life-threatening or major bleedinga, visceral ischaemia, or neurological impairment OR Distal embolization (non-cerebral) from a vascular source requiring surgery or resulting in amputation or irreversible end-organ damage OR The use of unplanned endovascular or surgical intervention associated with death, major bleeding, visceral ischaemia or neurological impairment OR Any new ipsilateral lower extremity ischaemia documented by patient symptoms, physical exam, and/or decreased or absent blood flow on lower extremity angiogram OR Surgery for access site-related nerve injury OR Permanent access site-related nerve injury
Major vascular complications	Access site or access-related vascular injury (dissection, stenosis, perforation, rupture, arterio-venous fistula, pseudoaneuysms, haematomas, percutaneous closure device failure) not leading to death, life-threatening or major bleedinga, visceral ischaemia, or neurological impairment OR Distal embolization treated with embolectomy and/or thrombectomy and not resulting in amputation or irreversible end-organ damage OR Any unplanned endovascular stenting or

	unplanned surgical intervention not meeting the criteria for a major vascular complication OR Vascular repair or the need for vascular repair (via surgery, ultrasound-guided compression, transcatheter embolization, or stent-graft)
Percutaneous closure device failure	Failure of a closure device to achieve haemostasis at the arteriotomy site leading to alternative treatment (other than manual compression or adjunctive endovascular ballooning)"
